# Fascia Is Able to Actively Contract and May Thereby Influence Musculoskeletal Dynamics: A Histochemical and Mechanographic Investigation

**DOI:** 10.3389/fphys.2019.00336

**Published:** 2019-04-02

**Authors:** Robert Schleip, Giulio Gabbiani, Jan Wilke, Ian Naylor, Boris Hinz, Adjo Zorn, Heike Jäger, Rainer Breul, Stephanie Schreiner, Werner Klingler

**Affiliations:** ^1^Department of Neuroanesthesiology, Neurosurgical Clinic, Ulm University, Günzburg, Germany; ^2^Department of Sports Medicine and Health Promotion, Friedrich Schiller University Jena, Jena, Germany; ^3^Fascia Research Group, Experimental Anesthesiology, Ulm University, Ulm, Germany; ^4^Department of Pathology and Immunology, Faculty of Medicine, University of Geneva, Geneva, Switzerland; ^5^Department of Sports Medicine, Institute of Sport Science, Goethe University Frankfurt, Frankfurt, Germany; ^6^School of Pharmacy, University of Bradford, Bradford, United Kingdom; ^7^Laboratory of Tissue Repair and Regeneration, Matrix Dynamics Group, University of Toronto, Toronto, ON, Canada; ^8^Division of Neurophysiology, Ulm University, Ulm, Germany; ^9^Anatomische Anstalt, Ludwig-Maximilians-Universität, München, Germany; ^10^Faculty of Health School – Clinical Sciences, Queensland University of Technology, Brisbane, QLD, Australia

**Keywords:** myofibroblasts, connective tissue, contractility, contracture, stiffness

## Abstract

Fascial tissues form a ubiquitous network throughout the whole body, which is usually regarded as a passive contributor to biomechanical behavior. We aimed to answer the question, whether fascia may possess the capacity for cellular contraction which, in turn, could play an active role in musculoskeletal mechanics. Human and rat fascial specimens from different body sites were investigated for the presence of myofibroblasts using immunohistochemical staining for α-smooth muscle actin (*n =* 31 donors, *n =* 20 animals). In addition, mechanographic force registrations were performed on isolated rat fascial tissues (*n* = 8 to *n* = 18), which had been exposed to pharmacological stimulants. The density of myofibroblasts was increased in the human lumbar fascia in comparison to fasciae from the two other regions examined in this study: fascia lata and plantar fascia [*H*(2) = 14.0, *p* < 0.01]. Mechanographic force measurements revealed contractions in response to stimulation by fetal bovine serum, the thromboxane A2 analog U46619, TGF-β1, and mepyramine, while challenge by botulinum toxin type C3–used as a Rho kinase inhibitor– provoked relaxation (*p <* 0.05). In contrast, fascial tissues were insensitive to angiotensin II and caffeine (*p* < 0.05). A positive correlation between myofibroblast density and contractile response was found (*r*_s_ = 0.83, *p* < 0.001). The hypothetical application of the registered forces to human lumbar tissues predicts a potential impact below the threshold for mechanical spinal stability but strong enough to possibly alter motoneuronal coordination in the lumbar region. It is concluded that tension of myofascial tissue is actively regulated by myofibroblasts with the potential to impact active musculoskeletal dynamics.

## Introduction

Compared with more discrete elements of the locomotor system — e.g., muscles, bones, disks, ligaments — the bag-like or planar collagenous connective tissue structures, commonly referred to as fascia, have received minor attention within musculoskeletal research (Grimm, 2007; Guimberteau et al., 2010; Kwong and Findley, 2014). Recent studies have elaborated the role of muscular fascia as essential force transmitter in muscular dynamics (Stecco et al., 2006, 2009; Huijing, 2009; Maas and Huijing, 2012; Pavan et al., 2015; Krause et al., 2016). However, fascia is usually considered as a relatively inert tissue that is assumed to serve a passive role only in musculoskeletal biomechanics.

In contrast to this common assumption there have been sporadic indications of a more active role of fascia due to an inherent ability to actively contract. These indications include the reported phenomenon of “ligament contraction” of human lumbar fascia in response to repeated isometric strain application *in vitro* (Yahia et al., 1993), the documented presence of interspersed cells with smooth muscle-like appearance in the human fascia cruris (Staubesand and Li, 1996; Staubesand et al., 1997; Bhattacharya et al., 2010), and the clinical experience of seemingly animated fascial tonus changes in response to fascia manipulation treatments frequently reported by manual therapists (Minasny, 2009) and acupuncturists (Langevin et al., 2001).

Sufficient evidence exists for the ability of fascial tissues to shorten over time frames of several days or more in certain pathologies, such as Palmar fibromatosis, Morbus Ledderhose, hypertrophic scars, and similar fascial fibrotic conditions (Desmoulière et al., 2005). It is generally assumed that the tissue shortening and stiffening observed in these pathological circumstances is driven by myofibroblasts (MFBs), and that the resulting tissue contracture is accomplished by an incremental combination of cellular contraction, collagen cross-linking and matrix remodeling in a slip and ratchet-like manner (Tomasek et al., 2002). It is, therefore, not surprising, that active tissue contractions—observed within time frames of several minutes post stimulation—have been successfully recorded *in vitro* with several of these pathologic tissues in response to pharmacological stimulation (Hurst et al., 1986; Naylor et al., 1994; Irwin et al., 1997; Raykha et al., 2013; Türker et al., 2013).

While these cells were mainly considered as an indicator for pathological conditions in the first years after the discovery of MFBs, subsequent studies have revealed their presence also in normal (i.e., non-pathological) ligaments (Murray and Spector, 1999), tendons (Ralphs et al., 2002), bronchial connective tissue (Kapanci et al., 1992), organ capsules (Chander et al., 1989), and several other collagenous connective tissues (Tomasek et al., 2002). Nevertheless, there have only been few explorations of contractile properties in normal fasciae. Preliminary investigations with a small sample of rat fascia pieces by Hinz et al. (2001) suggested an absence of MFBs and an inability to induce *in vitro* contractions in this tissue; while other studies described the presence of MFBs in the human deep fascia (Bhattacharya et al., 2010; Dawidowicz et al., 2015) and measurable tissue contractions of rat fascia in response to pharmacological MFB stimulation *in vitro* (Irwin et al., 1997; Pipelzadeh and Naylor, 1998; Schleip et al., 2016).

Based on this background, this study had three goals. First, a further investigation of the presence of MFBs in different fascial tissues. Second, an evaluation of their potential active responsiveness to pharmacological stimulation. Third, an estimation of resulting forces’ impact on musculoskeletal dynamics.

## Materials and Methods

### Study Design and Ethical Standard

The present study included three parts: an immunohistochemical analysis for the density of MFBs in human fasciae, a mechanographic investigation for potential contractile responses of fresh fascial tissues from rats in response to pharmacological stimulation, and a hypothetical calculation of the potential effect of fascial contraction forces on human musculoskeletal dynamics. All surgical and experimental procedures were in strict agreement with the guidelines and regulations of the Declaration of Helsinki and were approved by the ethical committee of the University of Ulm.

### Immunohistochemistry

Samples of human fasciae were taken as surplus tissue from autopsy studies of 28 individuals (*n =* 31, 25 males, 6 females, mean age 43 ± 37 years; range 17–91 years) at the institute for legal medicine of the Ludwig-Maximilian University Munich, Germany, or as surplus tissue from diagnostic muscle biopsies performed with informed consent at the department of applied physiology at Ulm University, Germany was used with informed consent and approval of the local ethics (*n =* 3). The procedure was anonymized and approved by the ethics committee of Ulm University, Germany (reference no. 37/97). Section sizes were approximately 8 mm × 8 mm × 0.5 mm from autopsy donors and 4 mm × 4 mm × 0.5 mm from biopsy donors. All autopsy and biopsy donors were Caucasian from the same geographical region in southern Germany. Donors with a known pathology affecting connective tissue morphology were neither included in the autopsy nor in the biopsy tissue collections. Sections were taken from the following sites: middle of plantar fascia, lumbar fascia (posterior lamina of posterior layer, 3–4 cm laterally of the spinous process of L3) and the fascia lata at the lateral thigh at midpoint between the greater trochanter and the fibular head. Sections from biopsy donors were taken from the described fascia lata location only. All sections were taken from the right-hand side of the body. For immunohistochemical comparison between rodent and human fasciae, 20 pieces of rat lumbar fasciae were randomly chosen from the rat tissue collection described under mechanographic methods.

Subsequent immunohistochemistry and quantification of α-smooth muscle actin (ASMA) density was conducted as described elsewhere (Schleip et al., 2018).

### Immunofluorescence

Paraffined sections for immunofluorescence were cut at 10 μm (to allow easier three-dimensional differentiation of MFBs from blood vessels). They were mounted on glass microscope slides (Superfrost^TM^/Plus, Thermo Fisher Scientific Inc., Pittsburgh, PA, United States). The slides were then deparaffinized with xylol and immersed in ethanol at decreasing concentrations (100, 100, 96, 80, 70, and 50%; each for 1 min) followed by immersion in distilled water for 3 min and washing 3 × 10 min with phosphate buffered saline (PBS). The sections were then incubated with mouse monoclonal primary antibody to ASMA-1 (mouse IgG2a mAB, clone 1A4, University of Geneva, Switzerland) (Skalli et al., 1986) at an IgG concentration of 5 μg/ml for 1 h, washed 3 × 10 min with PBS and subsequently probed with Alexa Flour 488 goat anti-mouse IgG (H+L) (Cat. No. A-11029, Molecular Probes, Inc., Eugene, OR, United States) PBS buffer with 0.2% BSA at room temperature in darkness (50 μl/coverslip). A DAPI stain was used to label nuclear DNA (Cat. No. 32670, Sigma-Aldrich, Taufkirchen, Germany). Sections were then washed 3 × 30 min with PBS, plus for 1 min with distilled water, both at room temperature in darkness and overlaid with one drop of polyvinyl alcohol as a mounting liquid.

Slides from immunofluorescence staining were imaged with a Bio-Rad Radiance 2000 confocal microscope (Bio-Rad Laboratories, Hemel Hempstead, United Kingdom) adapted to a Nikon ECLIPSE TE300 inverted Microscope (Nikon Corporation, Tokyo, Japan) with a 60x oil immersion lens (numerical aperture 1.4). Fluorescence was excited at 488 nm (Ar laser) and filtered by a 500 low pass emission filter. Vascular smooth muscle cells on the same slides were used as positive controls for immunostaining. Analysis of IF slides was used as additional reference only for a qualitative investigation about the distribution of positively stained areas in the examined tissue samples. Respective fascial tissue samples (*n =* 8) were randomly selected from the previously described human tissue collection.

### Mechanographic Investigation

Angiotensin II, caffeine, mepyramine, U46619, SQ-29548, Y-27632, and cytochalasin-D were obtained from Sigma-Aldrich, Steinheim, Germany. Recombinant TGF-β1 was obtained from Merck KGaA (Darmstadt, Germany). Fetal bovine serum (FBS) was obtained from Invitrogen (Karlsruhe, Germany), botulinum toxin type C3 from Pharm-Allergan GmbH (Ettlingen, Germany), and Krebs-Ringer (KR) solution from PAA Laboratories GmbH (Pasching, Austria).

**FIGURE 1:**
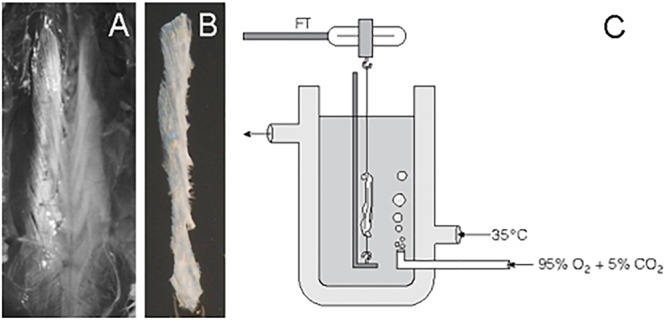
Handling of rat thoracolumbar fascia. After removal of skin and subcutaneous connective tissue, the underlying dense layer of thoracolumbar fascia was made accessible **(A)**. One long strip from the left side of the thoracolumbar spine (shown here in **B**) and also one from the other side were carefully dissected and cleaned of any attached muscle fibers. Some samples were then used for histochemical analysis while others were used for mechanographic registrations in an organ bath, as shown in **(C)**. Here the bath solution was aerated with carbogen. Through a double-walled container, the bath was kept at a constant temperature. The upper end of the tissue was connected with a force transducer (FT).

Tissue samples of thoracolumbar fascia were taken from 40 Wistar rats (22 male, 18 females, ages between 50 and 620 days, mean age 94 days, mean weight 345 g). Animals were sacrificed according to the local animal welfare guidelines of the University of Ulm. Between excision from the animal and final measurements the tissues were kept immersed in KR solution composed of (mM): 118 NaCl, 3.4 KCl, 0.8 MgSO_4_, 1.2 KH_2_PO_4_, 11.1 glucose, 25.0 NaHCO_3_, 2.5 CaCl_2_, pH 7.4; or were frequently sprayed-upon by KR solution (both at room temperature). A surgical knife was used to remove all visible muscle fibers from the fascia. This was controlled by inspection through a light microscope with 20x magnification. The thoracolumbar fascia was exposed, and a longitudinal piece was excised on the right side of the thoracolumbar spine, as well as a second piece on the left side (Figure 1A,B).

Before using a tissue for mechanographic measurements, each longitudinal piece was folded once, such that it became half as long but twice as thick. The new endings of this folded piece were then each fixed with a string end of mercerized cotton (diameter 160 μm, stiffness 12500 MPa). The effective sample size of such a folded piece had a length of 20–38 mm, a diameter of 1.5–3.5 mm; and a weight of 80–450 mg. The time between the death of the animal and recording of the last test with a given tissue was kept to below 8 h. The tissues were then suspended in an immersion organ bath filled with KR solution (Figure 1C). The solution in the bath was kept at a constant temperature of 35°C and bubbled with carbogen consisting of 95% O_2_ and 5% CO_2_ stabilizing neutral pH. The upper end of the tissue was connected with the free arm of an isometric force-voltage transducer (Model FT03, Grass Instruments, West Warwick, RI, United States), which was connected to a computer through a bridge amplifier and an analog-digital board (Digidata 1200B, Axon Instruments, Inc., Union City, CA, United States).

Four chambers were used simultaneously. A given test protocol was executed simultaneously in all four chambers, of which one chamber–the choice of which rotated between tests–was devoted to testing a non-viable control tissue. Pretreatment of this control tissue consisted of five cycles of deep freezing in liquid nitrogen followed by rapid thawing (Fuller, 1987; Schleip et al., 2016). The tissues were first suspended in their baths in a slack (non-extended) position. By slowly extending the tissue, the first point of a reversible force increase was defined as zero strain with zero force, provided this force increase was at least 10 nN and clearly repeatable by going back and forth across this zero point. From here, tissues were stretched up to 5% strain–a strain level shown before to lie below the magnitude required for imposing internal collagen fiber ruptures in the tissue (Yahia et al., 1993)–and then left at that strain for at least 45 min for equilibration before exposing them to pharmacological stimulation. All strain changes were conducted at a speed of 0.33%/s. All agents were adjusted to neutral pH before addition. In the few cases in which a tissue was exposed to multiple pharmacological tests, care was taken, that the remnants of the previous agent were washed off with at least two times the bath volume and that the original baseline of tissue tension had been (re)stabilized and kept constant for at least 15 min.

Fascia samples were examined for a potential force response to the following agents: FBS 30%, mepyramine at 10^-2^M, thromboxane analog U46619 at 10^-4^M, TGF-β1 at 15 ng/ml, botulinum toxin type C3 at 30 μg/ml, angiotensin II at 10^-2^M, and caffeine at 32 mM. In general, force registrations were concluded 1 h after substance addition. In case of an obvious stable baseline with no indication for any response to the pharmacological stimulation, some measurements were concluded beforehand, and their final force registrations were taken. Due to their observed slower response dynamics (Parizi et al., 2000; Kakudo et al., 2012), force registrations of the substances TGF-β1 and botulinum toxin type C3 were concluded after 3 h.

For the purpose of further exploration of the cellular dynamics involved in fascial contractility, some samples were preincubated for 30 min with the cytoskeletal inhibitor of actin polymerization cytochalasin D at 10^-6^M, with the thromboxane receptor antagonist SQ-29548 at 10^-6^M, or with the RHO/ROCK pathway inhibitor Y-27632 at 10^-5^M before U46619-induced contraction was measured.

Some of the animal tissues were not only used for mechanographic investigation but also for a subsequent immunohistochemical analysis (for immunohistochemical examination of possible ASMA density differences between the responder tissue samples and the samples that had proven as unresponsive in their mechanographic examinations).

### Force Calculation for Application to Human Biomechanics

Based on the mechanographic and immunohistochemical examinations, a hypothetical calculation of the potential contraction force of the intramuscular and extramuscular fasciae of the paraspinal musculature across the level of L3 in humans was conducted. This was done in three steps.

First, the potential force densities related to cross-sectional area (CSA) were calculated based on the maximum contractile force density observed in the mechanographic experiments with rats of our study. Alternatively, the cellular density of MFBs per mm^2^ from the person with the highest observed areal ASMA lumbar fascia density from our immunohistochemical investigations with humans was combined with the force of 4.1 μN/cell, reported as mean contraction of MFBs in the literature (Wrobel et al., 2002). Alternatively, this calculation also was conducted based on the cellular MFB density corresponding to the median areal lumbar fascia ASMA density.

The corresponding immunohistochemical estimation of the density of MFB cells was conducted in the following manner: As a rule, all visible nuclei were counted in a given field, which showed the stained fiber bundles arranged in extension of the long axis of the nucleus. Based on the three-dimensional spreading and polymorphic appearance of MFBs this procedure did not have the same degree of objectivity and precision as the digitally performed quantification of the aerial density. Therefore, counting was performed only on four histological samples representing the areal density of lumbar fascia from the person with the overall highest MFB density. Alternatively, a counting was conducted with four samples representing the median areal density found in human lumbar fascia.

Second, the related forces were applied to the CSA of relevant fascia in a horizontal cross-section at the level of L3. This was done by including the intramuscular and extramuscular fasciae of the paraspinal musculature. Values for the mean thickness and width of the respective extramuscular fasciae were adopted from Barker et al. (2007), consisting of posterior layer of lumbar fascia, middle layer and anterior layer. Values for the CSA of the paraspinal muscles (erector spinae, multifidus, psoas, quadratus lumborum) were taken from the magnetic resonance imaging measurements of Ranson et al. (2006). Using data from Kovanen et al. (1984) and Mackey et al. (2004) the proportion of intramuscular connective tissue within these tonic paraspinal muscles was estimated conservatively as 10%.

Third, potential contraction forces were then compared with the relevant threshold values reported in the literature for low back stability. As a threshold for mechanosensory stimulation the value of 35 mN–for afferent activation by pressure on ligaments (Johansson et al., 1991) was used as orientation. For mechanical joint stability, the value 18.2 N given by Cholewicki and McGill (1995) – minimal force required to prevent spinal buckling in neutral standing – was taken as the most suitable orientation.

### Statistical Analysis

Due to violations of the normality assumption, all data are reported as medians with 95% confidence intervals. Non-parametric testing was used to test for systematic differences. Unless indicated otherwise, the significance level for all analyses was set to *p* = 0.05, the employed software was IBM SPSS Statistics 22.0 for Windows (IBM, United States).

#### Cell Density

The mean numbers of cells counted in the lumbar fascia, fascia lata and plantar fascia were compared using the Kruskal–Wallis-test for independent samples. In case of significance, *post hoc* Mann–Whitney *U* tests, adjusted for multiple comparisons (Bonferoni–Holm correction), were conducted. To examine the relationship between cell density and force developed upon stimulation (see below), Spearman’s Rho correlation coefficient (r_s_) was calculated.

#### Mechanographic Investigations

The Wilcoxon signed-rank test was used to detect possible changes of the contraction state (force registration following stimulation with angiotensin II, caffeine, mepyramine, FBS, and U46619) in dependent samples. Mann–Whitney-*U* tests were performed to reveal the systematic differences in independent samples (stimulation with TGF-β1 and botulinum toxin type C3 versus control). In the latter, relative changes instead of absolute values were used if a baseline difference (*p <* 0.01) was found.

## Results

### Presence of Myofibroblasts in Human Fascial Tissues

**FIGURE 2:**
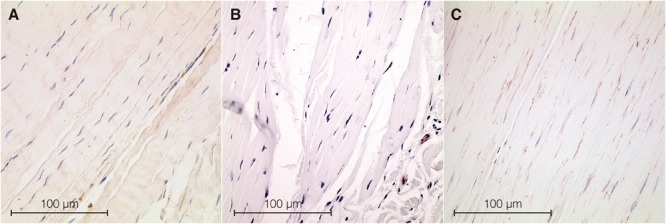
Histological sections from samples of fascia. ASMA positive stress fiber bundles—used as a marker for MFBs—were stained in dark brown, while cell nuclei were stained in dark blue. **(A)** Section from rat lumbar fascia. **(B)** Section from human fascia lata with a very low MFB density. **(C)** Section from human lumbar fascia. Microscopic inspection shows obvious differences in ASMA density.

ASMA stress fiber bundles, indicative of the presence of MFBs, were found in all examined human tissues (Figure 2). Cell density differed significantly between body sites [*H*(2) = 14.0, *p* < 0.01]. In the human lumbar fascia [median 1.52% (IQR 0.17–4.89%), *n* = 12], it was found to be considerably higher than in the human plantar fascia and the fascia lata [0% (0–0%, *n* = 11, *p* = 0.003) versus 0% (0–0.03%, *n* = 12, *p* = 0.003)]. When compared to the rat specimens, the density in human lumbar fascia showed a statistical trend toward being higher (*p* = 0.059, median human lumbar fascia 1.52%, IQR: 0.16–5.58%, rat lumbar fascia: 0.95%, IQR: 0.01–0.40%).

**FIGURE 3:**
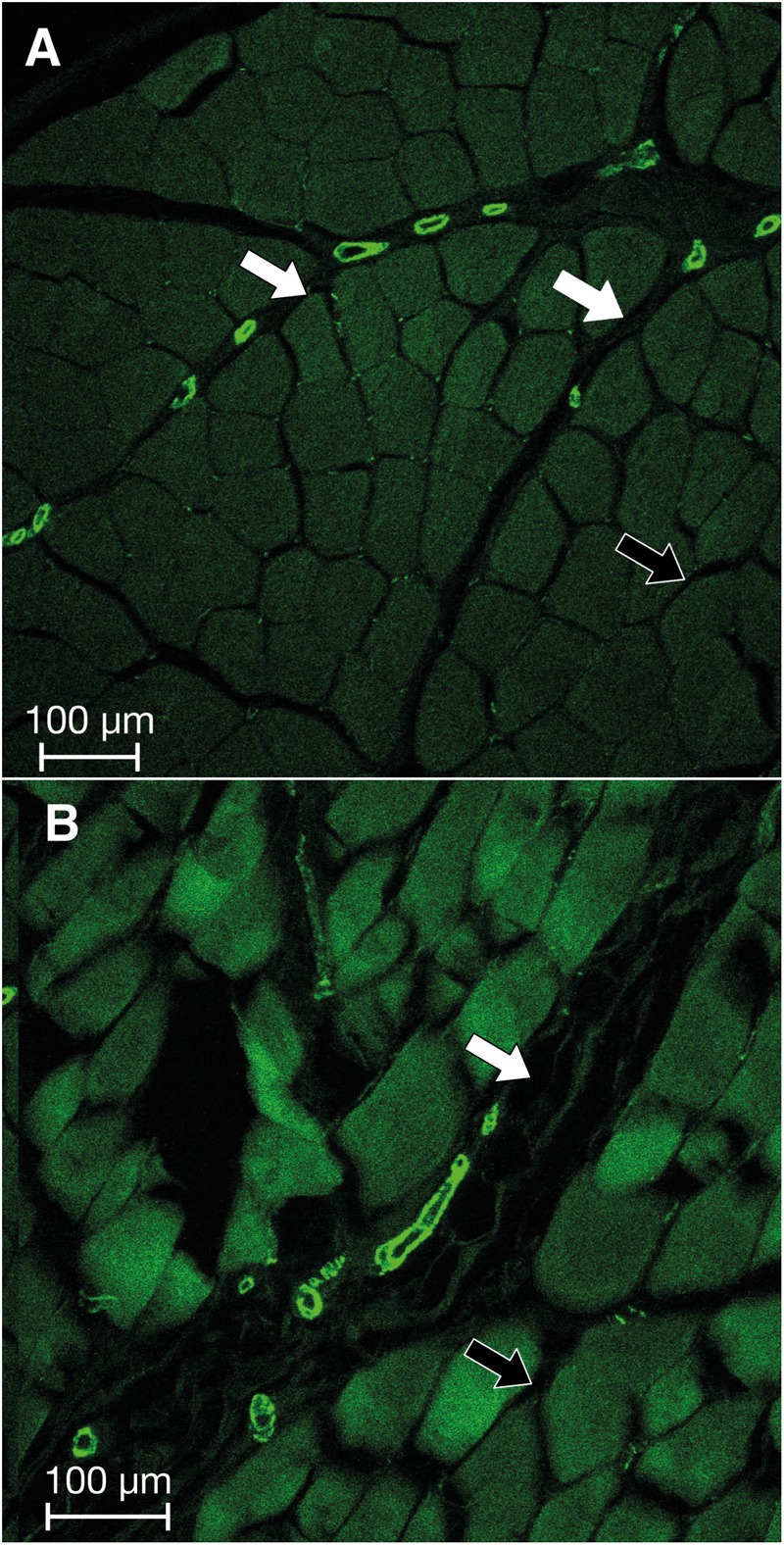
Immunofluorescence imaging of two representative sections of intramuscular fascia from the human lumbar region. Bright green: elements that are positively stained for the presence of ASMA. Note the apparently increased presence of MFBs in the perimysial zones (white arrows) as opposed to endomysial zones (black arrows) in both sections **(A,B)**.

Within the human lumbar fascia samples there was a trend toward higher MFB density in older donors, as expressed in a higher density in donors above 60 years (median 4.40% (IQR 0.36–6.38%), *n* = 5) compared with those under 30 years [median 2.26% (IQR 1.12–6.65%), *n* = 3], although this trend was not significant. Congruently, the overall correlation between MFB density in human lumbar fascia and donor age was not significant. No age-related trends were recognizable within the other two human tissue regions or within all human tissues. Within the rat lumbar fascia samples there was a moderate positive correlation between MFB density and age (*r*s = 0.60, *p* < 0.007), which was also expressed in a trend toward a higher MFB density in animals aged over 150 days [median 1.67% (IQR 0.46%–2.92%), *n* = 6] compared with those under 100 days [median 0.03% (IQR 0.00–0.09%), *n* = 11], although this difference was not significant. On an observational level, a surprising trend toward frequent high MFB density areas in the perimysium was noticed (see Figure 3).

**FIGURE 4:**
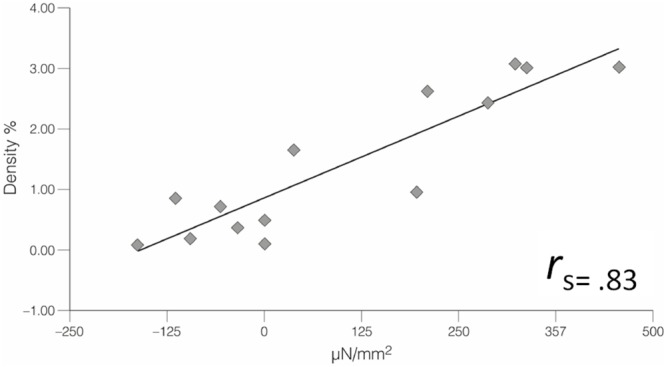
The MFB density of several samples of rat lumbar fascia was assessed (via immunostaining for ASMA) subsequent to their mechanographic examination in an organ bath environment. Statistical analysis revealed a strong positive correlation between the two factors, where higher MFB density was associated with more forceful contractile response (*n* = 14).

In general, the mechanographic stimulation revealed a strong positive correlation between cell density and contractile response (*r*_s_ = 0.83, *p* < 0.001, see Figure 4).

### Mechanographic Force Registrations With Rodent Fasciae

**FIGURE 5:**
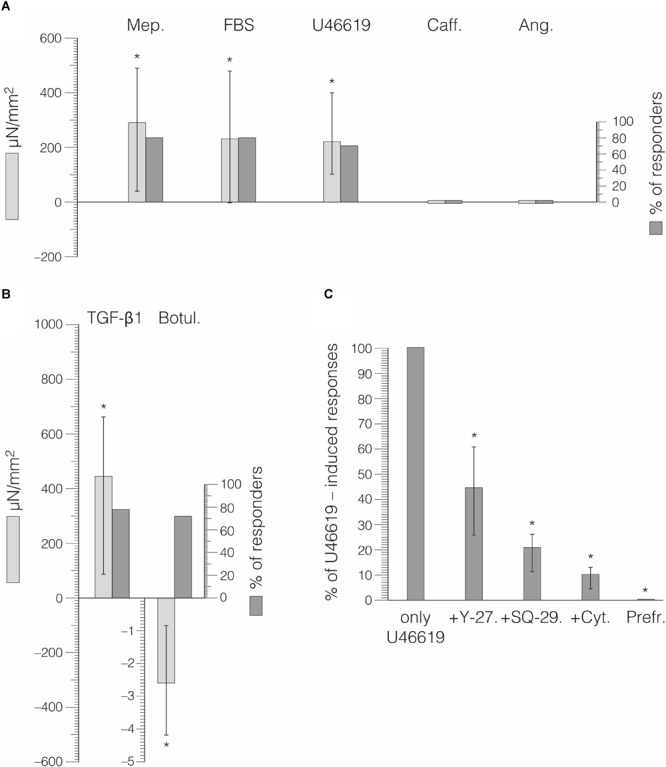
Force responses of rat lumbar fascia samples to exposure to different pharmacological agents. Error bars indicate interquartile range and ^∗^ the total range. **(A)** Percentage of responders shown and their mean force responses during 30 min of substance exposure. Mep, mepyramin; Caff, caffeine; Ang, angiotensin II. **(B)** Responses to TGF-β1 and botulinum toxin type C3 occurred during much longer time periods. **(C)** Incubation with specific inhibitor substances prior to stimulation with U46619 led to the reduced force responses—compared with stimulation by U46619 alone—shown here. Repeated preparatory cycles of freezing and rapid thawing completely abolished force response.

Neither stimulation with caffeine nor with angiotensin II yielded any clear force response (*n* = 8, *n* = 9, *p* > 0.05). The force responses following the application of the other substances are shown in Table 1 and Figure 5A,B. In brief, TGF-β1 yielded a clear contractile response when compared to untreated control samples (Hodges–Lehman estimate for the difference between relative prepost changes: 72.8%, 95% confidence interval: 42.6–157.9, *p* < 0.001). Mepyramine (median of relative prepost change: +6.9%, 95% CI: 4.0–14.7, *p* = 0.002), FBS (+6.9%, 95% CI: -2.4–11.5, *p* = 0.010), and the thromboxane analog U46619 (+6.3%, 95% CI: 0–14.5, *p* = 0.012) also led to significant force increases. In contrast to the other substances, botulinum toxin type C3 yielded a relaxation response when compared to untreated tissue samples (*p* < 0.001, Hodges–Lehman estimate for the difference between absolute prepost changes: 2.5 μN/mm^2^, 95% CI: 1.1–4.2).

**Table 1:** Overview of the main substances used in this investigation.

Substance	Dosage	Force maximum within interval of	Investigation type	*n* =	Response	Force change	Significance
Mepyramine	10^-2^ M	30 min	Unpaired	17	Contraction	+290 μN/mm^2^	*p* = 0.002
FBS	30%	30 min	Unpaired	11	Contraction	+230 μN/mm^2^	*p* = 0.010
U46619	10^-4^ M	30 min	Unpaired	14	Contraction	+220 μN/mm^2^	*p* = 0.012
Caffeine	32 × 10^-3^ M	30 min	Unpaired	8	None	N.A.	N.A.
Angiotensin II	10^-2^M	30 min	Unpaired	9	None	N.A.	N.A.
TGF-β1	15 ng/ml	3 h	Paired	18	Contraction^∗^	+445 μN/mm^2 ∗^	*p <* 0.001
Botulinum toxin type C3	30 μg/ml	3 h	Paired	17	Relaxation^∗^	–2.5 μN/mm^2 ∗^	*p <* 0.001

*Note that some of these mechanographic registrations involved the use of paired control samples from the same animal, which were exposed to exactly the same measuring procedure. Instead of the respective pharmacological agent, however, Krebs–Ringer solution was added into the organ baths of these control samples. In contrast, in the “unpaired” investigations all examined tissues were exposed to the pharmacological trigger. Previous investigation had shown that assessment times of 30 min were sufficient for measuring force responses with mepyramine, fetal bovine serum (FBS), and U46619. However, for TGF-β1 and botulinum toxin type C3, assessment times of 3 h had been chosen due to their more delayed and sustained response dynamics. Median values are shown for the force changes. ^∗^Relative force change compared with control samples.*

The force responses with FBS, U46619, and mepyramine usually happened within 30 min (time until apex between 20 and 40 min). With TGF-β1 and botulinum toxin type C3, the respective force changes were more delayed and long lasting, frequently continuing with small incremental changes at the end of the measurements (after 3 h).

**FIGURE 6:**
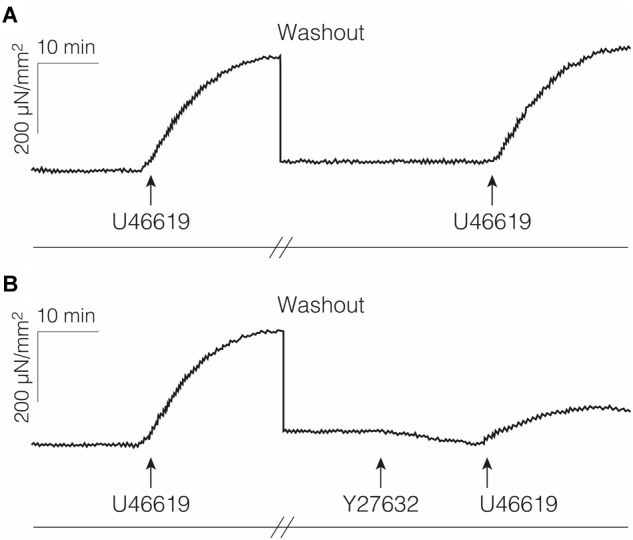
Examples of force responses with repeated stimulation. **(A)** Fascia sample was treated with U46619 and then washed out thoroughly for 1 h before repeated stimulation with the same agent. **(B)** Preincubation with the Rho-kinase inhibitor Y-27632 led to a reduced force increase in response to the 2nd application of U46619.

Pretreatments with the RHO/ROCK pathway inhibitor Y-27632 (*p* = 0.028), with the thromboxane A_2_ receptor antagonist SQ-29548 (*p* = 0.043) or cytochalasin, an inhibitor of actin polymerization (*p* = 0.018), lead to reduced force responses. Compared with the median force response to U46619, these pretreatments lead to force magnitudes that were reduced to the following proportions: Y-27632 median reduction to 44.7% (IQR 25.9–60.9%, *n* = 6), SQ-29548 reduction to 20.9% (IQR 11.2–26.3%, *n* = 5), and cytochalasin reduction to 10.1% (IQR 4.4–13.1%, *n* = 7). The corresponding results are shown in Figure 5C. The application of U46619 to the control samples that were pretreated by freeze-thaw cycles showed no force responses (*n* = 10). Figure 6 illustrates that the U46619 application is reversible. U46619 was applied twice with a washout step in-between and in which the addition of RHO/ROCK pathway inhibitor Y-27632 before the second application yielded a reduced force response.

### Potential Impact on Human Musculoskeletal Dynamics

The CSA of related extramuscular fasciae—as described in the method section—was found to be 199 mm^2^. Complemented by the CSA of paraspinal intramuscular fasciae (from erector spinae, psoas, and quadratus lumborum; with a combined muscular CSA of 12140 mm^2^), a total CSA of all intramuscular and extramuscular fasciae of 1413 mm^2^ was derived. Combined with the maximal contraction forces observed in our mechanographic force registrations (median force response to TGF-β1), a hypothetical contraction force of 0.95 N was gained.

In our second mode of calculation, we used the cellular density of MFBs as a basis of the potential contraction force. Here, we found an estimated MFB density of 167 cells/mm^2^ corresponding to the median ASMA density in the human lumbar fascia reported above. Alternatively, a cellular MFB density of 454 cells/mm^2^ was estimated for the person with the highest observed ASMA density in the lumbar fascia. Based on these densities, a predicted contraction force of 0.97 N was derived for median MFB density in the lumbar fascia and 2.63 N for the maximal MFB density found in our investigations.

None of these predicted forces lie above the threshold for exerting an impact on mechanical joint stability (Cholewicki and McGill, 1995). However, all of these predicted force values are above the much lower threshold for mechanosensory stimulation (Krauspe et al., 1992).

### Data Availability

The resulting immunohistological as well as mechanographic data are available upon request from the first author.

## Discussion

To our knowledge, this is the first study examining the question whether active cellular contractility of fascial tissues may be able to impact musculoskeletal dynamics. Our immunohistochemical plus mechanographic findings and related force calculations suggest this might be the case. Our findings suggest that, due to the contractile behavior of inherent MFBs, human lumbar fascia may be able to change its stiffness in a time frame of minutes to hours and thereby possibly affect motoneuronal coordination.

### Presence of MFBs in Fascial Tissues

The immunohistochemical findings of our study provide evidence for the existence of contractile cells, i.e., MFBs, in different fascial tissues. Yet, the related density appears to vary considerably between the examined tissues. The increased density of MFBs in human lumbar fascia—in comparison with all other human or murine tissue sources used in our examinations—suggests a possible association with the prevalence of myofascial pain in the human lumbar region (Furlan et al., 2012; Edwards et al., 2017). In fact, the presence of micro-injuries in human lumbar connective tissues and a resulting corrupted neuromuscular coordination in addition to other downstream effects have been suggested as novel explanations for some cases of low back pain (Panjabi, 2006; Langevin and Sherman, 2007; Willard et al., 2012; Karayannis et al., 2016). The finding of clearly nociceptive nerve endings in human lumbar fascia adds further support for that possibility (Tesarz et al., 2011; Mense and Hoheisel, 2016). In addition, a reduction in thoracolumbar shearing motion has been described in chronic low back pain patients compared with healthy controls (Langevin et al., 2011). The observed density of MFBs in human lumbar fascia in our study could possibly be associated with an augmented occurrence of (micro-) injuries and related cellular repair processes in human lumbar fasciae. It should be noted that our histological examination included fascial tissue sections from lumbar fascia, plantar fascia and fascia lata only. The choice of these limited tissue regions was influenced by the access options of our research group. Therefore, it will be interesting to include the investigation of potential regional MFB density differences between different fasciae from a larger regional variation in future studies.

The apparently increased density of MFBs in the perimysium (Figure 3), which was unexpectedly observed in this study, could be of clinical significance. In food science, meat toughness correlates with perimysial thickness (Bendall, 1967; Rowe, 1974) and aging recently has been found to be paralleled by an increased complexity in the organization of the perimysium (Mikkelsen et al., 2017). Since an augmented perimysial collagen density has been observed in tonic muscles as opposed to a lower density in more phasic muscles (An et al., 2014; Roy et al., 2018), this suggests that a related perimysial stiffening may indeed contribute to myofascial tonicity in human erector spinae muscles, and particularly to the deep multifidus layer (Mannion et al., 1997; MacDonald et al., 2006). In addition, several myofascial pathologies associated with increased myofascial stiffness are associated with changes in the perimysium (Williams and Goldspink, 1984; De Deyne et al., 2000; de Bruin et al., 2014). In contrast, these described changes have not been found in the endomysium. Similarly, aging tends to be associated with a decreased proportion of fast twitch muscle fibers as well as with an increased perimysial thickness (Nishimura, 2010; Csapo et al., 2014). It will be interesting to explore via future histological studies with larger sample sizes whether the trend toward a higher fascial MFB density in older patients, observed in part in our investigation, can be generally confirmed; and if so whether this increase may be associated with the larger total quantity of (micro)injuries in fascial tissues during previous life years, which may then induce an increased MFB density in the affected tissues. In short, our current findings suggest that the cellular density and activity of MFBs might play a contributory role in these tissue changes.

In a similar manner, our finding regarding the perimysium could add an interesting perspective to the hypothesis of Stecco et al. (2013)—that muscle spindle sensitivity is influenced by the stiffness of the perimysial connective tissue in which the spindle capsules are mostly embedded (Maier, 1999; Boyd-Clark et al., 2002). Based on the influence of spindle derived mechanosensation on alpha motorneuron activation this interaction may contribute to active muscle tonicity (Giuriati et al., 2018). According to this concept, several myofascial pain syndromes could be influenced by changes in perimysial stiffness (Stecco et al., 2016). Our finding suggests that the presence and activity of MFBs could be an important contributor in this interaction.

Contrary to our expectations, our data do not indicate a profound impact of age and sex on MFB density. If true, then the fascial stiffening observed in the temporal region of elderly subjects (Trindade et al., 2012) might rather be ascribed to changes of connective tissue architecture, the formation of collagenous cross-links, or changes of the hydration status. However, the large age range included in our histological examination (17–91 years, *n =* 31) should be regarded as a factor limiting respective interpretations. Regarding the role of sex, it should be underlined that our samples came predominantly from male body donors. Additional research is thus warranted to conclusively identify the potential influence of age and sex as well as the influence of other factors as modifiers of MFB density. The degree of physical activity and muscle volume represent two important candidates, which might both be positively correlated with the number of contractile cells (Szczodry et al., 2009).

### Contractile Behavior of Fascial Tissues

Our mechanographic measurements with rats showed a contractile response to several substances. Interestingly, the force registrations in our examinations revealed a large variation of the fascial tissues, even when stimulated with the same substance. This seems to fit to the finding of a similar large variation in MFB density found in our histological examination.

We suggest that these two features are related to each other, i.e., that the local density of MFBs seems to be a driving factor behind our observed tissue contractions. This would mean that if a tested tissue sample contained no or very few MFB cells only, then it expressed no or very tiny contractile forces in responses, whereas clear contractile responses were observed in samples with a higher MFB density. While our examinations allowed only a comparison between a small group of responder versus “non-responder” tissue samples (*n* = 7), the reported preliminary result from this comparison suggests that, in future, similar examinations could be useful and worthwhile to store and evaluate all used tissue samples from *in vitro* contraction tests for subsequent immunohistochemical analysis.

Our observed tissue contractions in response to TGF-β1 are not surprising, given that this substance has served as a successful contractile agent in cell culture examinations with MFBs and in scar tissues (Hinz et al., 2012). To our knowledge, this is the first experimental result indicating its capacity to induce contractile responses of normal fascial tissues. An almost linear increase in contraction force in response to TGF-β1 in cell culture has been demonstrated over a period of the first 20 h after substance addition (Brown et al., 2002). While this might possibly suggest up to six times higher contraction forces (compared with the 3 h period used in our measurements), further examinations are necessary for clarification to what extent a similar force increase over time exists in macroscopic tissues *in vitro* and *in vivo*. Given the reported signaling influence of the sympathetic nervous system on TGF-β1 expression (Bhowmick et al., 2009; Liao et al., 2014), our finding could possibly support the hypothesis of Staubesand and Li (1996, 1997), which proposed a close connection between fascial stiffness and sympathetic activation. In light of the large contribution of psychosocial factors in low back pain (Yang et al., 2016; Burgel and Elshatarat, 2017), this appears to represent an interesting direction of future research.

### Potential Impact on Musculoskeletal Dynamics

Our calculations of potential contractile forces *in vivo* predict a force range that seems insufficient for exerting a direct short-term effect (i.e., occurring within minutes to hours) on mechanical joint stability of the human spine, when using the threshold value of 18.2 N given by Cholewicki and McGill (1995) for the prevention of spinal buckling in neutral standing posture as orientation. It seems clear that potential short-term contractile forces of fascial tissues are at least two orders of magnitude below that of muscle tissue (of comparable CSA) and, therefore, can impose only minimal direct mechanical effects on the body. It is therefore indeed conceivable that active MFB contractions may have no significant effect on spinal stability or other important aspects of human biomechanics.

Nevertheless, our predicted fascial contraction forces in the human lumbar region are above the much lower threshold for influencing mechanosensation. According to Sjölander et al. (2002), any alteration of mechanosensation is potentially able to modify muscle coordination and reflex regulation of functional joint stability. Thus, we suggest that short-term stiffness changes in fasciae might possibly be sufficient to impact neuromuscular coordination. A temporarily increased fascial stiffness—e.g., due to an altered sympathetic nervous system activation and/or altered cytokine expression—might be able to modify or disrupt the accuracy of proprioception and coordination, which could possibly contribute to the likelihood of injuries and of subfailure injuries (Panjabi, 2006; Tong et al., 2017).

We suggest that a local and/or temporal increase in fascial contractility might also contribute to long-term tissue contracture, which includes matrix remodeling. This is based on the progress in the understanding of MFB biology since the original description of this cell type (Gabbiani et al., 1971). In particular, it has been shown that, differently from classical smooth muscle, the MFB exerts a relatively long-lasting Rho/ROCK/myosin light chain phosphatase pathway dependent contractile activity that eventually results in permanent tissue contracture (Bochaton-Piallat et al., 2016). Based on the lockstep ratchet model of MFB contraction (Tomasek et al., 2002; Follonier Castella et al., 2010; Hinz, 2013), tissue stiffening results from the contraction of single MFB cells and subsequent stabilization of tissues by secreted extracellular matrix molecules. This occurs as an incremental process in which long-lasting and strong RHO- and ROCK-dependent MFB contractions generate slack in collagen fibrils, whereas weak and short-ranged successive microcontractions (Ca^2+^) of the same cells remodel such relaxed fibrils. The new fibril configuration is then further stabilized, possibly by digestion of local collagen, deposition of new collagen fibrils, and cross-linking.

The combined action of this mechanism can generate severe tissue contractures (e.g., in frozen shoulder or Dupuytren’s contractures) of ∼1 cm per month (Follonier Castella et al., 2010). This suggests that density differences in MFBs—e.g., as have been explored in our study—and chronic alterations in sympathetic activation or other biochemical factors might not only lead to short-term changes which affect motoneuronal coordination but also may contribute long-term effects in the form of healthy well-regulated stiffness adaptations and pathologic contractures.

### Considerations for Low Back Stability

Our findings about an increased MFB density in human lumbar fascia together with these hypothetical force calculations suggest that the observed minor changes in lumbar fascia stiffness may possibly constitute a contributing factor to back stability and low back pain. The different layers of the human thoracolumbar fasciae have been shown to contribute significantly to trunk stability (Vleeming et al., 1995, 2014; Willard et al., 2012). Ultrasound examinations of the posterior layer of the thoracolumbar fascia indicated an increased thickness and reduced shearing motion of this fascial tissue in chronic low back pain patients (Langevin et al., 2009, 2011). A previous investigation already demonstrated two examples of lumbar fascia sections from low back pain patients with an augmented MFB density comparable to that found in frozen shoulder (Willard et al., 2012). Further research is warranted to delineate the potential relationships between fascial properties and low back stability and low back pain. Analyses of human lumbar fascia biopsies for the presence of MFBs and additional biochemical factors (Klingler et al., 2014) could serve as valuable examination pathway in this direction (Figure 2C).

MFB driven stiffness changes in lumbar fasciae may possibly also influence the complex dynamics of anticipatory adjustments which play an important role in human postural regulation (Park et al., 2014; Wang et al., 2018). In addition, minor fascial stiffness changes, as observed in our study, could be involved in – at least some cases of – the phenomenon of vertebral somatic dysfunction described as a minor intervertebral disorder in the osteopathic literature (Fryer et al., 2004; Ho, 2015; Tozzi, 2015). Further research, possibly using myometry, elastography or other stiffness oriented *in vivo* assessment methods, are warranted to investigate these potential influences (Giyoung et al., 2014; White et al., 2018).

### Methodological Considerations

Our force measurements involved small sample sizes only (see Table 1). We are not aware of any publication using mechanography in an organ bath environment with much larger sample sizes (see e.g., Irwin et al., 1997; Pipelzadeh and Naylor, 1998; Hinz et al., 2001; Moon et al., 2006; Hennenberg et al., 2018; Mader et al., 2018), which is reflective of the high demands in conducting these investigations. Nevertheless, it is important to point out that based on the small sample size all subsequent interpretations need to be treated with appropriate caution.

The choice of a non-viable control tissue that had been pretreated by freeze and thaw cycles had been previously introduced by Schleip et al. (2016). Such treatment had been shown to effectively kill all cells (Frank et al., 1988) while keeping passive viscoelastic tissue properties virtually unchanged (Smith et al., 1996; Moon et al., 2006). We suggest that the regular inclusion and comparison with these control tests in our examinations strengthens the assumption that our observed force changes in fascial tissues are indeed due to cellular responses.

The use of mepyramine as a stimulatory agent in our investigation deserves some reflection. Mepyramine is a histamine H1 receptor inverse antagonist. If used in supra-physiological concentrations (like in this study and in those of others), it can stimulate histaminic receptors (Naylor et al., 1994; Fitzsimons et al., 2004). While a therapeutic application of such high dosages on human patients is out of the question, the above reported contractile responses of fascial tissues to this substance suggest that H1 receptors on MFBs might be possible targets for future investigations exploring a therapeutic modulation of fascial contractility.

Botulinum toxin type C3—also known as C3-transferase—is one of the toxins produced by the bacterium Clostridium botulinum. In contrast to the more widely used botulinum toxin type A in modern medicine and cosmetics, it is not a neurotoxin but selectively ribosylates Rho GTPase in their effector-binding domain (Sekine et al., 1989). Previous cell culture examinations have shown that this substance can exert an inhibitory effect on the contractile activity of human MFBs (Parizi et al., 2000). To our knowledge, this study for the first time demonstrates that botulinum toxin type C3 is also capable of inhibiting contractile activity in fascial tissues. Since cell-permeability of this substance is a limiting factor, long incubation periods have been recommended (Fahrer et al., 2010). Based on this consideration and previous to our examinations it had therefore not been clear, whether this substance could be used in whole fascial tissues as an inhibitor of MFB contractility and/or as a relaxation inducer. The results of our *in vitro* experiments, in which stimulation of rat lumbar fascia produced a force decrease over a period of 3 h, tend to give support for the suggestion that therapeutic applications of botulinum toxin type C3 could be explored as a novel avenue in the treatment of fibrotic pathologies which are characterized by an increased contractile activity of MFBs (Namazi and Abdinejad, 2007).

No matter how carefully the fascial tissue bundles are dissected and prepared, some interspersed skeletal muscle fibers may still be included. It is, therefore, justified to consider the possibility that the fascial force changes observed in our tests may be due to tonus changes in such interspersed skeletal muscle fibers, rather than to tonus changes of MFBs. However, the fact that preincubation by Y-27632 inhibited potential contractile effects of stimulation by U46619 suggests a crucial role of Rho-kinase in tissue contractions, showing that the MFB contraction under our conditions was mainly calcium independent and fibroblast like. This assumption is corroborated by the results of control applications of U46619 or Y-27632 to comparable bundles of rat lumbar multifidus muscle tissue, which showed no detectable force changes of the bundles. In contrast, the subsequent application of 32 mM caffeine always elicited very clear contractions (*n* = 8, data not shown). In addition, the absence of any force response of fascial tissues in response to 32 mM caffeine in our findings contradicts a significant contribution of skeletal muscle fibers in the observed tissue contractions.

A similar argumentation can be considered in relation to the possibility that the observed tissue contractions could be due to the contraction of vascular smooth muscles cells within the tissue bundles. Here, the absence of a force response with angiotensin II suggests that vascular contractility did not play a significant role in our observed tissue contractions. In addition, the finding of a significantly higher MFB density in responder tissues compared with non-responder samples provides further corroboration for a strong dependency of our observed fascial contractions on the presence of MFBs.

While the histological examinations of this study were mostly performed with human tissues, the mechanographic investigations were exclusively conducted with fascial tissues from rats. It is therefore necessary to apply particular caution when combining and interpreting the different findings, In particular, the possibility cannot be excluded that the observed results may not represent the responsiveness of human fasciae. The following indications suggest that the responsiveness of human fascial tissues *in vitro* should be roughly comparable to the basic features observed in our murine tissues.

First, a previous investigation of Hoppe et al. (2014) included the demonstration of a sample of human vastus lateralis fascia expressing a clear contractile reaction in response to pharmacological stimulation, when examined in the same conditions. Second, our histological data indicate that the density of MFBs in human fascia is not less than that observed in comparable rat fascia. While this may be a demanding task for tissue acquisition and related ethics approval, we recommend that future studies should include *in vitro* contraction tests with surgical tissue samples from human donors.

### Perspectives and Significance

Our findings question the common clear distinction between active tissues and passive tissues in musculoskeletal dynamics (Panjabi, 1992). While the contraction forces observed in our study do not support a significant contribution of active fascial contractility in time frames of seconds (as are frequently considered, e.g., for locomotor dynamics), they suggest that active changes of fascial stiffness might play contributory roles to the motoneuronal coordination aspect of low back stability and other musculoskeletal parameters when viewed in a time-window of several minutes and longer. As some chronic disorders develop asymptomatically over a large time frame (Rio et al., 2014) and are characterized by increased tissue stiffness (Bolívar et al., 2013; Kuo et al., 2013), the potential contribution of fascial MFB activity merits further investigation.

## Ethics Statement

The study was carried out in accordance with the recommendations of Ethikkommission der Universität Ulm, Ulm, Germany. The protocol was approved by the Ethikkommission der Universität Ulm. All subjects gave written informed consent in accordance with the Declaration of Helsinki.

## Author Contributions

RS, RB, and WK contributed to the conception and design of the research. RS, SS, AZ, WK, and BH performed the experiments. RS, AZ, WK, and JW analyzed the data. RS, GG, IN, JW, WK, AZ, and RB interpreted the results of the experiments. RS, JW, WK, and BH prepared the figures. RS, HJ, JW, and WK drafted the manuscript. RS, JW, WK, and IN edited and revised the manuscript. RS, JW, WK, IN, AZ, SS, GG, HJ, RB, and BH approved the final version of the manuscript.

## Conflict of Interest Statement

The authors declare that the research was conducted in the absence of any commercial or financial relationships that could be construed as a potential conflict of interest.
